# Dendritic cells are defective in breast cancer patients: a potential role for polyamine in this immunodeficiency

**DOI:** 10.1186/bcr1001

**Published:** 2005-02-25

**Authors:** Alban Gervais, Jean Levêque, Françoise Bouet-Toussaint, Florence Burtin, Thierry Lesimple, Laurent Sulpice, Jean-Jacques Patard, Noelle Genetet, Véronique Catros-Quemener

**Affiliations:** 1 Groupe de Recherche en Thérapeutique AntiCancéreuse, UPRES 2261, Faculté de Médecine de Rennes, Rennes, France; 2 Service de Gynécologie, Centre Hospitalier Universitaire de Rennes, Hôpital Sud, Rennes, France; 3 Laboratoire de Cytogénétique et Biologie Cellulaire, Centre Hospitalier Universitaire de Rennes, Hôpital Pontchaillou, Rennes, France; 4 Laboratoire d'Anatomo-Pathologie, Centre Hospitalier Universitaire de Rennes, Hôpital Pontchaillou, Rennes, France; 5 Service d'Oncologie Médicale, Centre AntiCancéreux de Rennes, Avenue de la Bataille Flandres-Dunkerque, Rennes, France; 6 Departement de Chirurgie Viscérale, Centre Hospitalier Universitaire de Rennes, Hôpital Pontchaillou, Rennes, France; 7 Service d'Urologie, Centre Hospitalier Universitaire de Rennes, Hôpital Pontchaillou, Rennes, France; 8 Laboratoire d'Immunologie, Centre Hospitalier Universitaire de Rennes, Hôpital Pontchaillou, Rennes, France

## Abstract

**Introduction:**

Dendritic cells (DCs) are antigen-presenting cells that are currently employed in cancer clinical trials. However, it is not clear whether their ability to induce tumour-specific immune responses when they are isolated from cancer patients is reduced relative to their ability *in vivo*. We determined the phenotype and functional activity of DCs from cancer patients and investigated the effect of putrescine, a polyamine molecule that is released in large amounts by cancer cells and has been implicated in metastatic invasion, on DCs.

**Methods:**

The IL-4/GM-CSF (granulocyte–macrophage colony-stimulating factor) procedure for culturing blood monocyte-derived DCs was applied to cells from healthy donors and patients (17 with breast, 7 with colorectal and 10 with renal cell carcinoma). The same peroxide-treated tumour cells (M74 cell line) were used for DC pulsing. We investigated the effects of stimulation of autologous lymphocytes by DCs pulsed with treated tumour cells (DC-Tu), and cytolytic activity of T cells was determined in the same target cells.

**Results:**

Certain differences were observed between donors and breast cancer patients. The yield of DCs was dramatically weaker, and expression of MHC class II was lower and the percentage of HLA-DR^-^Lin^- ^cells higher in patients. Whatever combination of maturating agents was used, expression of markers of mature DCs was significantly lower in patients. Also, DCs from patients exhibited reduced ability to stimulate cytotoxic T lymphocytes. After DC-Tu stimulation, specific cytolytic activity was enhanced by up to 40% when DCs were from donors but only up to 10% when they were from patients. IFN-γ production was repeatedly found to be enhanced in donors but not in patients. By adding putrescine to DCs from donors, it was possible to enhance the HLA-DR^-^Lin^- ^cell percentage and to reduce the final cytolytic activity of lymphocytes after DC-Tu stimulation, mimicking defective DC function. These putrescine-induced deficiencies were reversed by treating DCs with all-*trans *retinoic acid.

**Conclusion:**

These data are consistent with blockade of antigen-presenting cells at an early stage of differentiation in patients with breast cancer. Putrescine released in the microenvironmement of DCs could be involved in this blockade. Use of all-*trans *retinoic acid treatment to reverse this blockade and favour *ex vivo *expansion of antigen-specific T lymphocytes is of real interest.

## Introduction

The role played by T cell mediated immunity in the control of tumour growth has been established over recent years. As a result, most immunization strategies adopted in clinical trials of cancer treatments have aimed at enhancing tumour antigen (TA)-specific cellular immunity. The induction and expansion of TA-specific T cells requires optimal antigen presentation and T-cell co-stimulation. Dendritic cells (DCs) are specialized antigen-presenting cells with a remarkable ability to stimulate naïve T lymphocytes and generate memory T lymphocytes [1]. However, objective response rates to vaccine or DC trials in cancer remain low [2]. Differentiation and maturation of DCs are important to their protective activity against tumour development [3]. Exposure to necrotic tumor cells can induce maturation of immunostimulatory DCs [4] but the involved mechanisms are still unresolved [5].

Cytotoxic T lymphocytes (CTLs) directed against tumour cells can be amplified *in vitro *with the use of DCs pulsed with treated tumour cells (DC-Tu) [6]. When assays were done with cells from healthy donors, DC-Tu stimulation repeatedly increased the final cytolytic activity of T cells more than twofold. However, we observed that a similar procedure applied to cells from cancer patients enhanced the final cytotoxic activity against autologous tumour only in half of the assays [6]. We noticed in these experiments that the final yield and phenotype of blood-derived myeloid immature DCs was heterogeneous in cancer patients [6]. These findings could be related to a relationship between immune suppression instilled during tumour development, as previously described by Kusmartsev and Gabrilovich [7], and increased production of immature myeloid cells in patients with advanced cancers [8].

Our aim in the present study was to detail the differences in characteristics of DCs between patients with cancer and healthy donors. We investigated blood cells from patients with breast, colorectal, or renal carcinoma and compared them, using the same assays, with cells from healthy donors. DCs were obtained from peripheral blood [9] and matured using various cocktails combining proinflammatory cytokines and danger or co-stimulating signals that are known for their ability to induce a T-helper-1 phenotype [3,10]. Tumour cells were from the M74 melanoma cell line in all of the assays. Treatment of tumour cells was done for induction of late apoptosis (postapoptotic necrotic tumour cells) [11]. Necrotic cells were chosen for DC pulsing, in accordance with previous reports [5,12,13] and preliminary experiments by our group that demonstrated that processing and cross-presentation of TA led to specific CTL responses in DCs pulsed under these conditions (Gervais A, unpublished data).

The ultimate mechanisms by which DC deficiency is established are not understood. The tumour microenvironment is rich in growth factors and molecules that are able to modulate the immune response of the host. Polyamines, which are conducive to proliferation and metastatic invasion, are synthesized in large amounts by tumour cells [14]. A therapeutic strategy combining inhibition of all cellular and exogenous sources of polyamines has been evaluated in several murine tumour models, with positive findings [15]. However, the role played by polyamines in immune processes is poorly understood [16,17]. Nevertheless, our group showed that polyamine deprivation can prevent the development of *in vivo *tumour-induced immunosuppression [18]. In the present study, the hypothesis that putrescine is involved in immunodeficiency was tested by investigating the effects of putrescine on functional activity of DCs from donors. This treatment was able to mimic the abnormalities observed in DCs from patients with breast cancer.

## Materials and methods

### Patients

Thirty-four patients with histologically confirmed cancer were enroled in the study. Seventeen (age 47–76 years) had breast cancer: 13 had infiltrating ductal carcinoma (grade I-III; Elston Ellis grading); one was invasive lobular carcinoma (grade III); one was mixed ductal-lobular carcinoma (grade I); and two were in situ ductal carcinomas (low and high grade). Seven (age 33–86 years) had colorectal cancer (stage 2–4 adenocarcinoma) and 10 (age 42–78 years) had renal cell carcinoma (Fuhrman grade III clear cell carcinoma). Patients were newly diagnosed and peripheral blood samples were collected at the time of initial surgery, with no prior therapy. The study was approved by the regional ethics committee (Comité Consultatif de Protection des Personnes dans la Recherche Biomédicale de Rennes, Rennes, France). Eleven healthy volunteers (HLA-A2) served as control individuals (Etablissement Français du Sang, Rennes, France).

### Isolation of cells from peripheral blood

Cells were centrifuged by applying a density gradient (UNISEP^®^; Novamed, Jerusalem, Israel). Mononuclear cells (MNCs) were frozen in human serum albumin and 10% dimethyl sulphoxide until use for DC and lymphocyte preparation.

### Tumour cell treatment

The HLA-A2 MelanA-Mart1 expressing M74 melanoma cell line was used for both antigen DC pulsing and as a target for evaluation of specific cytotoxic activity. This cell line and the K562 natural killer (NK) cell-sensitive erythroleukaemia cell line were maintained in RPMI 1640 medium (Eurobio, Les Ulis, France) containing 10% foetal calf serum (Gibco Invitrogen Life Technologies, Cergy Pontoise, France), 1 mmol/l L-glutamine, 50 μg/ml streptomycin and 50 IU/ml penicillin (ICN Biomedicals, Aurora, OH, USA).

M74 cells were used for TA DC pulsing following necrosis-inducing treatment. The method was adapted from that presented by Lennon and coworkers [11]. Briefly, cells were treated with hydrogen peroxide 10 μmol/l (Sigma-Aldrich, Saint Quentin-Fallavier, France) for 3 consecutive days. Supernatant cells were collected each day, pooled and kept at 4°C. Collected cells (M74 per) were used for DC pulsing (DC-Tu_per_).

Treated tumour cells were examined for degree of apoptosis and secondary necrosis using a standard fluorescence-activated cell sorting assay (Annexin V-FITC detection kit; Immunotech, Marseille, France), which detects binding of annexin V (A) and propidium iodide inclusion/exclusion. With 10 μmol/l peroxide, 16% of the collected cells were apoptotic (annexin V positive/propidium iodide negative) and 50% were in a state of postapoptotic necrosis (annexin V positive/propidium iodide positive).

### Culture of dendritic cells

DCs were prepared from MNCs (from patients or healthy donors) in accordance with the method described by Sallusto and Lanzavecchia [9]. Briefly, 10 × 10^6 ^MNCs were seeded in 5 ml serum-free X-Vivo 10 medium (Biowhittaker, Walkersville, MD, USA) in a 25 cm^2 ^culture flask (Cellstar^®^; Greiner Labortechnik, Frickenhausen, Germany). Nonadherent cells were collected after 2 hours for lymphocyte culture. The remaining adherent cells were cultured in DC medium: serum free X-Vivo 10 medium supplemented with 10% AB serum (EFS de Rennes, Rennes, France), 10 μg/ml steptomycin and 100 IU/ml penicillin. Granulocyte–macrophage colony-stimulating factor (GM-CSF) 1000 IU/ml (Leucomax 400™; Novartis/Shering Plough, Huningue, France, Switzerland) and 400 IU/ml IL-4 (Promokine; Promocell, Heidelberg, Germany) were added on days 0, 2 and 5 of culture. After 7 days nonadherent cells (immature DCs) were collected and added to peroxide-treated M74 cells (ratio 1:10) for antigen processing. After 18 hours of contact, supernatant cells (DC-Tu_per_) were added to autologous lymphocytes (DC:lymphocyte ratio 1:100) for lymphocyte stimulation. DCs were phenotypically characterized before lymphocyte stimulation (day 8).

For maturation assays, immature DCs were seeded in DC medium (density 10^6 ^cells/ml) in 24-well plates (Falcon^®^; Becton Dickinson, Franklin Lakes, NJ, USA) and maturating agents were immediately added. Three different maturation cocktails were evaluated. The first (cocktail A) was a combination of tumour necrosis factor-α (25 ng/ml; Pharmingen, San Diego, CA, USA), lipopolysaccharide (10 μg/ml; Sigma-Aldrich) and CD40L (0.4 μg/ml; Alexis Biochemicals, QBiogene, Illkirch, France). The second (cocktail B) was a combination of IL-1β (10 ng/ml), IL-6 (1000 U/ml), tumour necrosis factor-α (10 ng/ml; R&D systems, Lille, France) and prostaglandin E_2 _(1 μg/ml; Sigma-Aldrich). The third (cocktail C) was a combination of bacterial extracts (1 μg/ml; Ribomunyl^®^; Pierre Fabre Médicament, Boulogne, France) and IFN-γ (1000 U/ml; Imukin^®^, Boehringer Ingelheim, Reims, France). After 18 hours of contact, supernatant cells (matured DCs) were collected and phenotypically characterized (day 8).

### Phagocytic activity of dendritic cells

Phagocytic activity was evaluated using FITC-labelled opsonized bacteria (*Escherichia coli*; Phago Test^®^; OrpeGen Pharma, Heidelberg, Germany). Immature DCs from patients or donors and opsonized bacteria were co-cultured for 2 hours at 37°C and their internalization was evaluated by flow cytometry, in accordance with the manufacturer's recommendations. Controls were run at +4°C.

### IL-10 and IL-12 production

Measurements of IL-10 and IL-12 were done in supernatants of immature DCs and mature DCs after 72 hours in culture at 37°C. Assays were done using enzyme-linked immunosorbent assay methods according to the manufacturer's instructions (Ready-set-Go^®^; eBioscience, San Diego, CA, USA) and were performed in duplicate.

### Dendritic cell treatments

Immature DCs were treated for 18 hours with 10 mmol/l putrescine (1,4-diaminobutane dihydrochloride; Sigma-Aldrich) before phenotype or functional activity were measured. Because it was reported that all-*trans *retinoic acid (ATRA) can reduce the number of immature myeloid cells and favour their differentiation into DCs [8], putrescine-treated immature DCs were added with ATRA (Sigma-Aldrich); 1 μmol/l ATRA was added each day for 5 days, in accordance with the procedure described by Almand and coworkers [8]. Phenotype and functional analyses were conducted after 5 days with or without ATRA treatment.

### Lymphocyte culture

Lymphocytes were cultured from MNCs in lymphocyte medium: RPMI 1640 containing 10% AB serum, 1 mmol/l L-glutamine, 2% pyruvate, 1% nonessential amino acids (Bioproducts, Gagny, France), 100 μg/ml streptomycin, 100 IU/ml penicillin and 150 IU/ml IL-2 (Proleukin^®^; Chiron, Suresnes, France). After 8 days in culture (density 10^6 ^cells/ml), lymphocytes were stimulated with DC-Tu_per_. Number, phenotypic and functional characteristics of lymphocytes were evaluated 7 days after DC-Tu_per _stimulation. Viability was evaluated using the trypan blue exclusion test. Controls were performed with nonstimulated lymphocytes.

### Flow cytometry analysis

Cells (10^5^) were suspended in phosphate-buffered saline supplemented with 0.5% bovine serum albumin and labelled for characterization of lymphocyte or DC phenotype by incubation at 4°C for 30 min with the following PE-, FITC-, or PC5-conjugated antibodies and corresponding isotypes: anti-CD3 (clone UCTH1), anti-CD4 (13B8.2), anti-CD8 (B9 11), anti-CD25 (B1.49.9), anti-CD40 (mAb 89), anti-CD56 (NKH-1), anti-αβ-TCR (BMA 031), anti-γδ-TCR (immu 510), anti-CD80 (MAB 104), anti-CD83 (HB15A) and anti-CD152 (CTLA-4; after saponin permeabilization) from Immunotech (Marseille, France); anti-CD11c (S-HCL-3), anti-HLA-DR (L243) and Lin1 cocktail (anti-CD3, anti-CD14, anti-CD16, anti-CD19, anti-CD20 and anti-CD56) from Becton Dickinson/Pharmingen (CA, USA); and CD86 (BU63) from Immunotech (Oxford, UK). Cells were washed and suspended in 250 μl phosphate-buffered saline added with 0.3% formol. CD4^+^CD25^+^CTLA4^+ ^was considered to be the T regulatory cell phenotype, in accordance with the findings of Jonuleit and coworkers [19]. Data analysis was performed using a FACScan flow cytometer (Becton Dickinson).

### Cytotoxicity assays

T-cell mediated cytotoxicity was tested in triplicate with a standard ^51^Cr release assay. The assays were conducted in U-bottomed microtitre plates. Depending on the assays, target cells were M74 tumour cell line or K562 cells pulsed with ^51^Cr ([^51^Cr]sodium chromate; Amersham Life Sciences, Buckinghamshire, England) for 1 hour.

A total of 5000 target cells/well were mixed with effector cells (ratio of effector to target cells 50:1) and incubated for 4 hours. Chromium release was assessed in culture supernatants using a γ-counter (Topcount, Packard Instrument, Rungis, France). Specific release was calculated as follows: ([mean experimental counts/min - mean spontaneous counts/min]/ [mean maximum counts/min - mean spontaneous counts/min]) × 100.

### IFN-γ production

Responder cells were evaluated for their production of IFN-γ in response to contact with antigenic cells. Analyses were performed 8 days after stimulation with DC-Tu_per_. Briefly 2 × 10^5 ^M74 cells were seeded in 24-well plates for 12 hours. The supernatant was discarded before adding 10^5 ^lymphocytes in a final volume of 500 μl of medium without IL-2. The plates were then incubated at 37°C for 72 hours and IFN-γ was measured in supernatant using enzyme-linked immunosorbent assay methods, in accordance with the manufacturer's instructions (Ready-set-Go^®^; eBioscience). Duplicate wells were run for each assay.

### Statistical analysis

Each assay was repeated with at least three different donors or patients. The nonparametric Mann–Whitney rank test was used for statistical analysis.

## Results

### Yield and characteristics of dendritic cells

**Figure 1:**
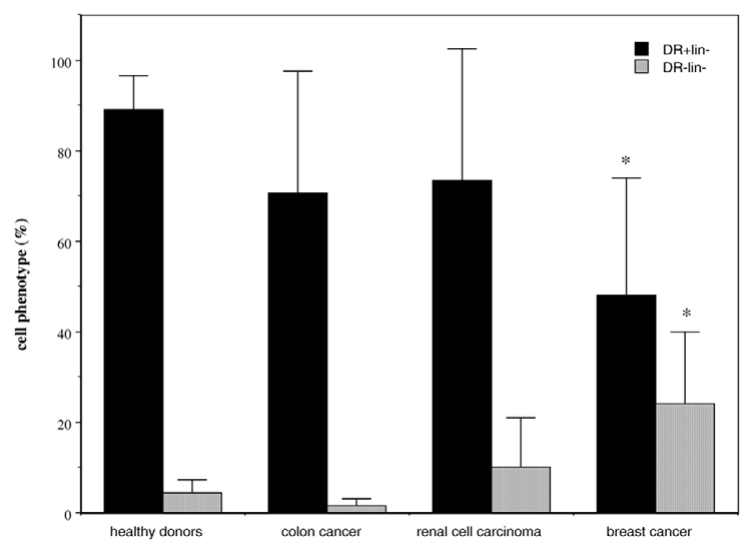
Phenotype of cells collected after immature dendritic cell preparation procedure in cancer patients. Peripheral blood mononuclear cells from healthy donors (*n *= 11) or patients with colorectal cancer (*n *= 7), renal cell carcinoma (*n *= 10), or breast cancer (*n *= 15) were depleted of lymphocytes (2-hour adherence step) and cultured for 7 days in the presence of granulocyte–macrophage colony-stimulating factor and IL-4. Data are expressed as the percentage of cells (with standard error) expressing the HLA-DR^+^Lin^- ^and HLA-DR^-^Lin^- ^phenotype. *P < 0.01 versus healthy donors.

After 7 days in culture with GM-CSF and IL-4, a mean of 12.5% of the blood MNCs from healthy donors differentiated into immature DCs (Table 1). These cells were predominantly HLA-DR^+^CD11c^+ ^(94 ± 4%) and CD11c^+^Lin^- ^(87 ± 13 %), which is characteristic of myeloid DCs. The yield of immature DCs was reduced after the same procedure was conducted in MNCs from patients with cancer (Table 1), and this reduction was significant for MNCs from patients with breast cancer. These patients had normal blood monocyte counts (0.44 ± 0.09 Giga/l). Furthermore, immature DCs prepared from peripheral blood MNCs from breast cancer patients expressed high levels of CD11c (79 ± 14% CD11c^+^Lin^-^), but large individual differences in HLA-DR expression were recorded. The percentages of HLA-DR^+^Lin^- ^cells were significantly reduced and HLA-DR^-^Lin^- ^significantly increased in patients with breast cancer (Fig. 1).

This latter observation led us to focus our DC investigations on breast cancer patients. On comparing the expressions of CD40, CD83 and CD86 on immature DCs between healthy donors (*n *= 6) and patients with breast cancer (*n *= 10), no significant differences were observed (CD40: 87 ± 7% versus 78.5 ± 16%, respectively; CD83: 6 ± 6% versus 10 ± 10%; and CD86: 82 ± 14% versus 69 ± 22%). For patients with colorectal cancer or renal cell carcinoma, percentages of HLA-DR^-^Lin^- ^cells in DCs were not significantly enhanced when compared with those for healthy donors (Fig. 1).

The phagocytic capacity of immature DCs from breast cancer patients was similar to that of immature DCs from healthy donors (respectively; at 37°C: 42 ± 9% and 44 ± 15%; and at 4°C: 5 ± 1% and 2 ± 1%). Mean fluorescence intensity after 2 hours of co-culture with FITC-labelled bacteria was 653 ± 56 and 656 ± 30 for patients and healthy donors, respectively.

### Maturation of dendritic cells

**Figure 2:**
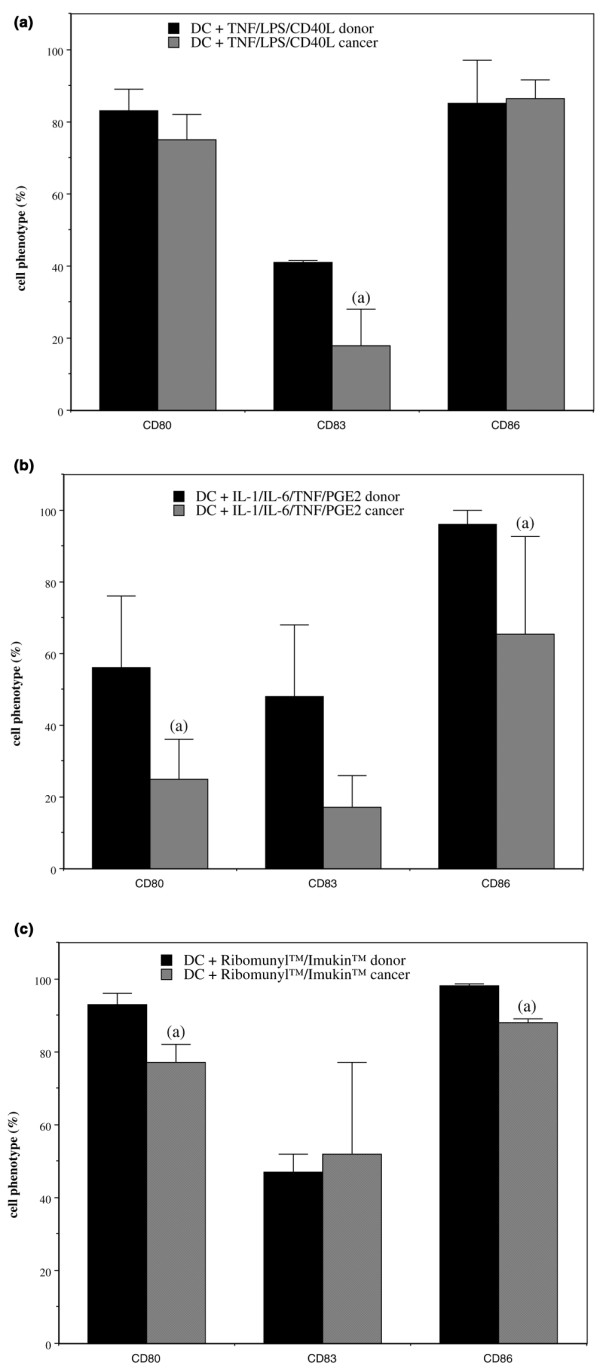
Maturation of dendritic cells (DCs) from healthy donors or from breast cancer patients. Data are expressed as the percentage of the cells (with standard error) expressing the CD80, CD83 and CD86 surface markers after treatment of immature DCs with a combination of maturating agents: **(a) **tumour necrosis factor (TNF)-α/lipopolysaccharide (LPS)/CD40L (*n *= 3); **(b) **IL-1β/IL-6/TNF-α/prostaglandin (PG)E_2 _(*n *= 4–5); and **(c) **Ribomunyl^®^/Imukin^® ^(*n *= 3). ^a^Different from corresponding donors in each individual assay.

For donors and patients, the three maturation cocktails induced significant increases in expression of CD80 and CD83 markers (Table 2, Fig. 2). However, the level of maturation reached by DCs was weaker for patients than for donors; whatever the combination of maturating agents used, we observed lesser expression of mature DC markers in patients (Fig. 2). For donors, high percentages of CD86- and CD40-expressing cells were similarly observed in immature and mature DCs (Table 2). These markers were heterogeneously expressed in patients. IL-10 and IL-12 production by immature DCs was similar in cells from donors and patients (Table 2). Interestingly, maturation induced by Ribomunyl^®^/Imukin^® ^stimulated IL-12 production more for DCs from patients than for DCs from donors (Table 2).

### Dendritic cell mediated T-cell stimulation

**Figure 3:**
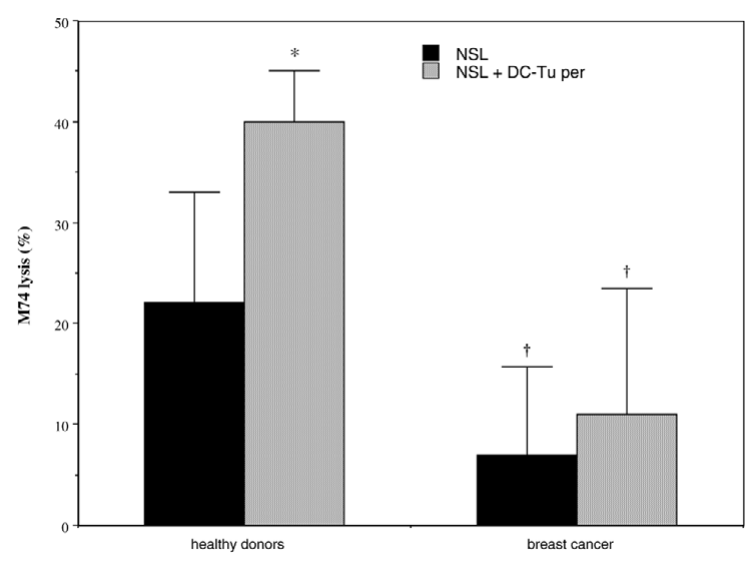
Cytolytic activity of lymphocytes from healthy donors or breast cancer patients against the M74 cell line. Lymphocytes are from donors (*n *= 5) or from cancer patients (*n *= 6), and were stimulated with autologous immature dendritic cells (DCs) pulsed with peroxide-treated M74 cells (DC-Tu_per_). Controls are nonstimulated lymphocytes (NSL). Values are expressed as cytolytic activity (with standard error) against M74 target cell line. *P < 0.03 versus NSL; ^†^*P *< 0.05 versus corresponding donors.

When lymphocytes were subjected to DC-Tu_per _stimulation, expansion was observed. The Expanding Index was not significantly greater in healthy donors (7.5 ± 2; *n *= 7) than in cancer patients (5 ± 2.5; *n *= 5). However, contrary to our observations in healthy donors, the cytolytic activity of lymphocytes against the M74 cell line was not significantly enhanced after DC-Tu_per _stimulation for breast cancer patients (Fig. 3), which indicates that DC-mediated T-cell stimulation was unsuccessful in the patients.

In addition, the basic cytotoxic activity of lymphocytes against the M74 cell line was significantly less for cancer patients than for healthy donors (Fig. 3). The differences persisted after DC-Tu_per _stimulation. In contrast, nonspecific lysis of the natural killer (NK) cell sensitive K562 cell line remained unchanged after DC-Tu_per _stimulation both for donors and for patients (respectively: from 51 ± 38% to 51 ± 25% lysis and from 19 ± 18% to 23 ± 23% lysis). Taken together, these observations suggest that TA-specific T cells were induced in donors but not in all of the breast cancer patients. No correlation could be established between reduced cytotoxic activity of lymphocytes from patients (against M74 or NK-sensitive cell lines) and percentage of regulatory T cells in the bulk (0.17 ± 0.14% CD4^+^CD25^+^CTLA4^+ ^cells; *n *= 11).

### Lymphocyte phenotype and IFN-γ production

Basic IFN-γ production in response to contact with M74 cells was similarly heterogeneous for lymphocytes from patients and those from donors. After DC-Tu_per _stimulation, enhancement in IFN-γ production – a marker of T-helper-1 response – was consistently observed in lymphocytes from healthy donors (Table 3); this was in contrast to patients, for whom enhancement was seen only in two out of six assays. Considerable reduction in IFN-γ production was seen in lymphocytes from patient S137, indicating that autologous DCs were not immunogenic in the assay (Table 3). Phenotypic characterization revealed 71% HLA-DR^-^Lin^- ^cells in DCs from patient S137.

Lymphocytes from donors and cancer patients were of similar phenotype after 15 days in culture with 150 UI IL-2 (Table 4). Of the cells, 70% were T lymphocytes and more than 50% were CD8^+ ^T cells. Single stimulation with DC-Tu_per _did not changed the respective percentages of CD4^+ ^T cell, CD8^+ ^T cell, or γδ T cell subpopulations. In addition, the percentages of regulatory T cells remained similar after DC-Tu_per _stimulation, at 0–0.5% of cells.

### Influence of putrescine treatment on dendritic cell phenotype

**Figure 4:**
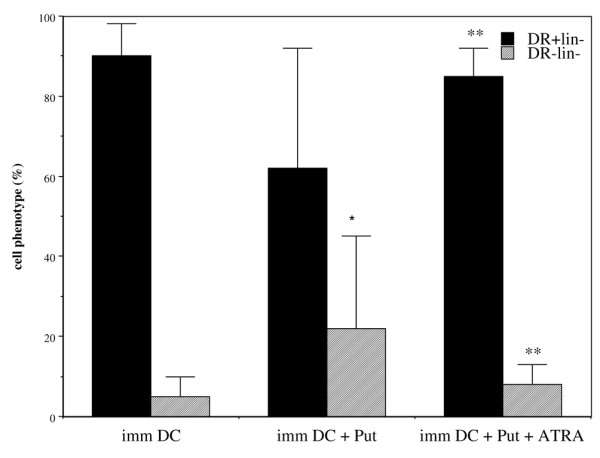
Effect of putrescine and all-*trans *retinoic acid (ATRA) on immature dendritic cell (DC) phenotype. Cells were collected after immature DC preparation procedure (imm DC; *n *= 11) and treated with 10 mmol/l putrescine (Put; *n *= 10). To putrescine-treated DCs was added 1 μmol/l ATRA (Put + ATRA; *n *= 5). Data are expressed as percentage of cells (with standard error) expressing the HLA-DR^+^Lin^- ^and HLA-DR^-^Lin^- ^phenotypes. *P < 0.01 versus imm DCs; **P < 0.02 versus putrescine-treated imm DCs.

As shown in Fig. 1, of cells prepared from MNCs from donors according to the classic procedure for preparing immature DCs, a mean of only 4.6% had the HLA-DR^-^Lin^- ^phenotype. An 18-hour treatment with 10 mmol/l putrescine increased this percentage to 29.5% (Fig. 4). Expression of other surface markers (CD40, CD80, CD83 and CD86) was not changed by putrescine treatment (data not shown). Putrescine was internalized by DCs because intracellular putrescine concentrations were dramatically enhanced after treatment (data not shown).

When DCs were treated daily for 5 days with 1 μmol/l ATRA, the phenotypic change induced by putrescine was reversed (Fig. 4).

### Putrescine-treated dendritic cells are defective in their ability to stimulate T cells

**Figure 5:**
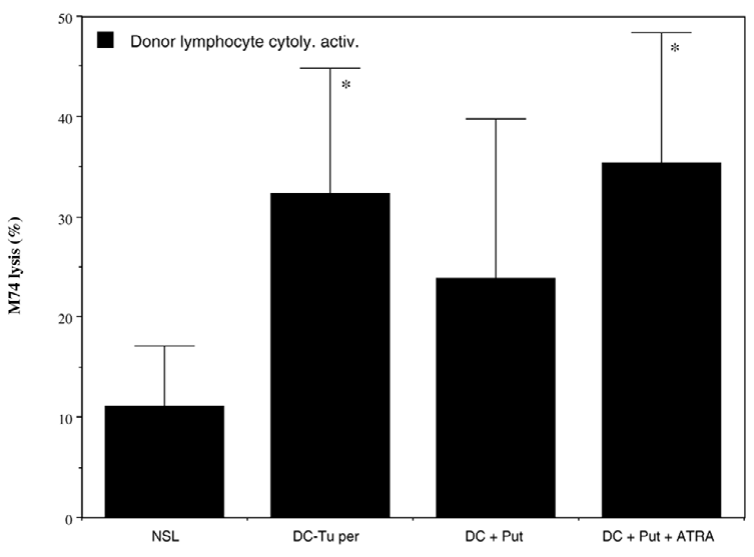
Cytolytic activity of lymphocytes stimulated with putrescine and all-*trans *retinoic acid (ATRA) treated dendritic cells (DCs). Immature DCs were from healthy donors and were treated with putrescine (Put) with or without ATRA before DCs pulsed with treated tumour cells (DC-Tu_per_) preparation. Autologous lymphocytes were stimulated with DC-Tu_per_, and data are expressed as cytolytic activity (with standard error) against M74 target cell line. Presented data are from seven different donors. Controls are nonstimulated lymphocytes (NSL). Decrease in M74 lysis was repeatedly observed for each of the donors in DC + Put compared with DC, and increased in DC + Put + ATRA as compared with DC + Put. *P < 0.05 versus NSL.

When DCs from donors were treated with putrescine, their ability to stimulate autologous T cells was significantly reduced. Following the DC-Tu_per _stimulation procedure, the Expanding Index of T cells declined by a mean 30 ± 11% when DCs were treated with putrescine (3.3 ± 1.9 versus 4.6 ± 2.5). In addition, specific cytolytic activity of DC-Tu_per_-stimulated lymphocytes was decreased when DCs were treated with putrescine (Fig. 5). This reduction was consistently observed in all donors (*n *= 6). Treatment with ATRA reversed this putrescine-induced deficiency in DCs and restored cytolytic activity against M74 cells to normal values. A similar increase was repeatedly observed for all donors. These changes were not observed when the K562 target cells were used for nonspecific NK-type cytolytic activity (data not shown).

## Discussion

In recent years several groups have described defective immune function in tumour-bearing animals [18,20,21] and in cancer patients [8,22,23]. Of note, it was reported that factors produced by tumour cells could influence differentiation of DCs from CD34^+ ^progenitors, and that low concentrations of IL-4 could reverse the inhibitory effect of cancer cell conditioned medium, at least in terms of phenotype and some functional differentiation of DCs [22]. We show here that, even in the presence of IL-4, differences in differentiation of circulating monocytes into DCs persisted in cancer patients as compared with healthy donors. Using the classic procedure of blood monocyte derived DC culture (in the presence of IL-4 and GM-CSF), the *ex vivo *yield of DCs was found to be significantly reduced in patients with cancer, particularly in those with breast cancer. Furthermore, the phenotype of collected cells using this procedure was different in patients with breast cancer. Expression of MHC class II (HLA-DR^+^Lin^- ^cells) was found to be lower and the percentage of HLA-DR^-^Lin^- ^to be higher than in donors. In contrast, these subpopulations were not significantly modified in patients with colon or renal cell carcinoma.

Whatever combination of maturating agents was used, significantly lower expressions of mature DC markers were observed in patients with breast cancer. Maturation induced by Ribomunyl^®^/Imukin^® ^resulted in lower expressions of CD80 and CD86 in patients than in donors, but, interestingly, it also resulted in greater production of IL-12.

Other groups have reported that, in breast cancer patients, monocyte-derived DCs have substantially lower level of expression of HLA-DR than do DCs isolated from control donors, leading to a reduced ability to stimulate allogenic and Flu-specific T-cell responses [8]. We confirm here that DCs from such patients not only exhibit low expression of MHC class II but they also have reduced ability to cross-prime exogenous antigens. Stimulation of CTLs by pulsed DCs was less efficient in patients than in donors. In a similar procedure for lymphocyte stimulation, using the same antigen preparation (peroxide-treated tumour cells) and tumour target (M74 cell line), we repeatedly observed defective stimulation when DCs were from patients with breast cancer. In general, the natural cytolytic activity of lymphocytes against the M74 or NK target cell line was found to be lower in patients than in donors. Unlike donors, patients were not selected for their expression of HLA-A2 class I molecules. This could represent an advantage in terms of CTL activation, but the opposite was observed. Cytolytic activity was enhanced by up to 40% when DCs were from donors but only up to 10% when they were from patients.

IFN-γ production after DC-Tu stimulation was repeatedly found to be enhanced in donors. In contrast, nonspecific lysis of the NK-sensitive K562 cell line was the same after DC-Tu_per _stimulation, clearly indicating that in donors DC-Tu_per _stimulation induced TA-specific T cells. Induction of TA-specific T cells did not occur in all cancer patients. Blockade of DCs at an early stage in differentiation could be responsible for this inconsistency between patients. For example, in patient S137 a high percentage of HLA-DR^-^Lin^- ^cells was observed in DCs, and concomitantly IFN-γ lymphocyte production was reduced twofold after DC-Tu stimulation, indicating that DCs were not only nonimmunogenic but were actually tolerogenic in this patient. However, the percentage of regulatory T cells was not changed after DC-Tu stimulation (<0.1% in S137). Correlation could not be demonstrated in this study between clinical grade of disease and HLA-DR^-^Lin^- ^DC phenotype. Defective function and poor ability of immature DCs to mature in some patients could represent an additional reason why DC cell therapy in cancer patients has, contrary to expectations, not yet yielded significant clinical responses [2].

Defective DC function can be mimicked by adding putrescine to the culture medium of DCs from healthy donors. The percentage of cells with HLA-DR^-^Lin^- ^phenotype was found to be enhanced after putrescine treatment. In addition, expansion and final cytolytic activity of lymphocytes was reduced following the DC-Tu_per _stimulation procedure, leading us to conclude that adding putrescine to the microenvironment of antigen-presenting cells blocks their ability to cross-prime exogenous antigens efficiently, indicating a reduction in their immunogenic function. It was reported by other authors that spermine, another polyamine, is responsible for severe inhibition in proinflammatoty cytokine synthesis when added to cultures of human peripheral blood MNCs stimulated with lipopolysaccharide [24].

Our group previously established that polyamine deprivation leads to significant reduction in tumour growth in murine experimental models. Consistent with that effect, an enhancement in CD8^+ ^T lymphocytes was observed in the spleens of the animals [18]. With similar experimental tumours we observed that combining polyamine deprivation with cyclophosphamide, which is known to downregulate regulatory T cells [25], enhances macrophage tumouricidal activity, indicating that the two treatments have synergistic effects [26].

In addition, breast cancer tissues are characterized by high polyamine levels. In a study including 174 patients with invasive breast cancer [27], a correlation was established between enhancement of putrescine and spermidine levels and tumour aggressiveness. Taken together, these observations led to the conclusion that putrescine release by tumour cells may be involved in the defective DC function observed in breast cancer patients. Interestingly, we showed in the present study that *in vitro *treatment of DCs with ATRA could reverse the putrescine-induced deficiency in DC function. ATRA and retinoic derivatives are known to influence DC differentiation, favouring a T-helper-1 response [28]. Further investigations are needed to detail the mechanism underlying the reversal in putrescine-induced deficiency in DC function. Nevertheless, use of ATRA treatment to initiate TA-specific CTL expansion in cancer patients could be of particular interest.

## Conclusion

Taken together, our findings are in agreement with those from Gabrilovitch and coworkers [7] on the contribution of immature myeloid DCs to cancer-induced immunosuppression – a mechanism that is involved in the escape of tumours from immune system control. Breast cancers are known frequently to over-express several TAs, such as carcinoembryonic antigen, MUC1, HER2/neu, P53 and members of the MAGE family, but little is known about detection of pre-existing T-cell responses, and the rationale for initiating vaccination strategies remains to be fully established. Nevertheless, a phase I clinical trial using vaccine prepared by fusing autologous tumor and DCs (32 patients included) [29] found that two patients with metastatic breast cancer exhibited disease regression. Our opinion is that future vaccination strategies could be improved in view of the present data. Procedures (established with cells from donors) must be adapted to the characteristics of the patient's DCs. One simple treatment would be use ATRA to reverse blockade of DC function. The Ribomunyl^®^/Imukin^® ^combination has demonstrated ability to induce DC maturation.

## Abbreviations

ATRA = all-*trans *retinoic acid; CTL = cytolytic T lymphocyte; DC = dendritic cell; DC-Tu = DCs pulsed with treated tumour cells; GM-CSF = granulocyte–macrophage colony-stimulating factor; IFN = interferon; IL = interleukin; MHC = major histocompatibility complex; MNC = mononuclear cell; NK = natural killer; TA = tumour antigen.

## Competing interests

The author(s) declare that they have no competing interests.

## Authors’ contributions

AG carried out the DC-Tu preparation, lymphocyte stimulation procedures and measurements of functional activities, and participated in writing the manuscript. JL selected the patients with breast cancer and took biological samples. FB-T participated in the design and coordination of the study. FB carried out anatomo-pathological examinations. TL participated in drawing blood samples in breast patients. LS selected the patients with colonic carcinoma and took biological samples. J-JP selected the patients with renal cell carcinoma and took biological samples. NG carried out cytometric analyses. VC-Q conceived the study, participated in its design and coordination, and wrote the manuscript.

## Figures and Tables

**Table 1:** Dendritic cell yield in patients with cancer and healthy donors

DC source	DC yield (%)
Healthy donors (*n *= 8)	12.5 ± 5.0
Patients with colorectal cancer (*n *= 4)	5.9 ± 2.6
Patients with breast cancer (*n *= 6)	3.1 ± 1.2*

Peripheric blood mononuclear cells (PBMCs) were cultured with granulocyte–macrophage colony-stimulating factor and IL-4. Data presented are the dendritic cell (DC) yield after 7 days: number of CD11c^+^Lin^- ^cells/number of PBMCs at day 0. DCs were prepared from blood of healthy donors, patients with colorectal cancer, or patients with breast cancer. *P < 0.01 versus donors.

**Table 2:** Cell surface phenotype of immature and mature dendritic cells from breast cancer patients

Donors/patients	Immature DCs/mature DCs		
Healthy donors			

Donor	M13	N14	015
CD40	79/95	80/97	84/96
CD80	0.1/90	1/97	18/91
CD83	2/52	8/48	19/40
CD86	85/98	68/98	57/97
IL-10	43/46	47/214	89/112
IL-12	5/400	7/29	5/182

Patients with breast cancer			

Patient	S219	S221	S222
CD40	91/93	42/86	77/86
CD80	3/84	4/76	4.5/72
CD83	8/17	14/66	14/72
CD86	87/89	25/86	68/89
IL-10	46/0	57/132	30/63
IL-12	bdl/2154	bdl/1280	bdl/1261

Data are expressed as the percentage of HLA-DR^+ ^cells expressing CD40, CD80, CD83 and CD86, and IL-10 and IL-12 production by dendritic cells (DCs) before and after maturation with cocktail C: Ribomunyl^®^/Imukin^®^. Data are individual values from patients S219, S221 and S222. Controls are from three different donors (M13, N14, O15). bdl, below the detection limit; DC, dendritic cell.

**Table 3:** Lymphocyte IFN-γ production after DC-Tu_per _stimulation

Donors/patients	IFN-γ (pg/ml)	
	
	NSL	NSL + DC-Tu_per_
Donors		

C3	516	1581
G7	52	257
H8	17	133
B2	0	8

Breast cancer patients		

S97	18	420
S101	12	908
S137	339	165
S108	418	176
S126	13	13
S94	13	10

IFN-γ production (pg/ml) was measured in response to tumour cells. Controls were nonstimulated lymphocytes (NSL). Data are individual values from four different donors and six breast cancer patients. DC-Tu, dendritic cells pulsed with treated tumour cells.

**Table 4:** Phenotype of DC-Tu_per _stimulated lymphocytes

Phenotype (%)	Donor		Breast cancer	
	
	NSL	NSL + DC-Tu_per_	NSL	NSL + DC-Tu_per_
CD3^+^CD56^-^	73 ± 30	76 ± 24	75 ± 17	73 ± 15
CD3^-^CD56^+^	6 + 8	5 ± 6	10 ± 13	5 ± 5
CD3^+^CD56^+^	18 ± 21	18 ± 18	11 ± 5	20 ± 13
CD4^+ ^T cell	28 ± 30	24 ± 27	29 ± 21	31 ± 22
CD8^+ ^T cell	51 ± 21	57 ± 18	51 ± 19	59 ± 18
TCR α/β	75 ± 23	77 ± 23	70 ± 20	83 ± 12
TCR γ/δ	14 ± 15	13 ± 18	14 ± 11	11 ± 12

Cells are from healthy donors (*n *= 3) or breast cancer patients (n = 6). The percentage of positive cells for lymphocyte markers was measured in a 99% CD45^+ ^population. Data are expressed as mean ± standard deviation. DC-Tu, dendritic cells pulsed with treated tumour cells; NSL, nonstimulated lymphocytes.
